# Characterization of the rumen and fecal microbiome in bloated and non-bloated cattle grazing alfalfa pastures and subjected to bloat prevention strategies

**DOI:** 10.1038/s41598-019-41017-3

**Published:** 2019-03-12

**Authors:** E. Azad, H. Derakhshani, R. J. Forster, R. J. Gruninger, S. Acharya, T. A. McAllister, E. Khafipour

**Affiliations:** 1 Department of Animal Science, University of Manitoba, Winnipeg, MB Canada; 2 Agriculture and Agri-Food Canada, Lethbridge Research Center, Lethbridge, AB Canada

## Abstract

Frothy bloat is an often fatal digestive disorder of cattle grazing alfalfa pastures. The aim of this study was to investigate ruminal and fecal microbiota dynamics associated with development of alfalfa-induced frothy bloat and to further explore how bloat prevention strategies influence the composition of these microbial communities. In a 3 × 3 crossover experiment, twelve rumen-cannulated steers were sequentially subjected to: (1) pure alfalfa pasture, (2) pure alfalfa pasture supplemented with the pluronic detergent ALFASURE, and (3) alfalfa – sainfoin mixed pasture. Eleven out of 12 steers in pure alfalfa pasture developed clinical bloat, whereas ALFASURE treatment prevented the development of bloat in all 12 steers and alfalfa – sainfoin prevented bloat in 5 out of 11 steers. Development of bloat was associated with considerable shifts in the microbiota profile of rumen contents. In particular, the microbiota of solid rumen contents from bloated steers contained higher species richness and diversity. *Streptococcus*, *Succinivibrio* and unclassified Myxococcales were enriched in the rumen microbiota of bloated steers, whereas *Fibrobacter* and *Ruminococcus* were overrepresented in the rumen contents of non-bloated steers. Our results provide novel insights into bloat-associated shifts in the composition and predicted functional properties of the rumen microbiota of cattle grazing alfalfa pasture.

## Introduction

Frothy bloat is an often-fatal digestive disorder of cattle grazing on highly digestible legumes or wheat pastures^1^. Although ruminants themselves lack the required enzymatic repertoire to harvest energy from lignocellulose-rich plant material, microbial symbionts inhabiting the rumen ecosystem can sequentially breakdown and ferment plant cell wall carbohydrates and thus support the energy requirement of ruminants^2^. Legume forages contain high concentrations of digestible proteins, which upon release into the rumen result in the rapid proliferation of ruminal microbes and an increase in fermentative activities and gas production^1^. This rapid proliferation of microbes, in particular the formation of bacterial biofilms, results in the production of an excessive amount of bacterial slime (i.e. exopolysaccharides) which increase the viscosity of the rumen contents and entrap fermentation gases in a stable foam that cannot be expelled by eructation^1,3^. In addition, legumes also possess glycosides (i.e. saponins) with foaming properties that can play a secondary role in development of bloat^4^. Among all North American forage legumes, alfalfa (*Medicago sativa*) has been the most widely adopted due to its high nutritive quality and yield. However, the occurrence of bloat is a major limitation to its greater use in grazing systems^5^. Several preventive strategies have been deployed in an attempt to mitigate bloat in cattle grazing alfalfa pastures. These include breeding for bloat-reduced alfalfa (AC Grazeland^6^), the use of alcohol ethoxylate/pluronic detergents, such as ALFASURE^7^, and the inclusion of non-bloating legumes such as sainfoin (*Onobrychis viciifolia*) in mixed legume pastures^8,9^.

Understanding the contribution of rumen microbiota to the development of frothy bloat is of high importance for implementing effective preventive strategies against this digestive disorder. Howarth *et al*.^10^ proposed that highly digestible high-protein forages, such as alfalfa, clover, and vegetative wheat result in the proliferation of certain ruminal microbial populations^1^ that promote bloat. To date, most of our knowledge regarding the contribution of rumen microbes to bloat is limited to either culture-based studies^11,12^, or targeted amplification and quantification (qPCR) of classical rumen bacteria^13,14^. However, the majority of microbes inhabiting the rumen ecosystem are unculturable^15^ or lack the genomic information for targeted amplification and therefore have likely been overlooked as important components within the rumen microbiome that contribute to frothy bloat. More recently, Pitta *et al*.^16^ used high-throughput sequencing to explore the association of the rumen microbiome with development of frothy bloat in steers grazing vegetative wheat pastures. They observed that wheat-induced frothy bloat was associated with an enrichment of several bacterial genera within the phylum Firmicutes, including *Clostridium*, *Eubacterium*, and *Butyrivibrio*, and an underrepresentation of genes encoding for oligosaccharidases. While this study generated valuable insight into the role of the rumen microbiome in development of wheat-induced frothy bloat, there remains a knowledge gap regarding the contribution of the rumen microbiota to alfalfa-induced frothy bloat.

The main objectives of our study were to (a) determine changes in the composition and functional properties of the rumen microbiota underlying the development of alfalfa-induced frothy bloat, (b) explore the response of rumen microbiota to dietary interventions to prevent bloat, including the grazing of alfalfa-sainfoin mixed pastures and the addition of the pluronic detergent ALFASURE, and (c) to investigate the degree that fecal microbiota can be influenced by these bloat prevention strategies.

## Results

Following introduction to pure alfalfa pasture, 11 out of 12 steers developed clinical bloat. In contrast, ALFASURE completely prevented bloat and alfalfa – sainfoin was moderately effective as 5 out of 11 steers bloated (Table 1).

**Table 1:** Summary of study design and incidences of bloat.

Sequence^a^	Period 1		Period 2		Period 3	
	Treatment^b^	Bloat status^c^	Treatment	Bloat status	Treatment	Bloat status
S1	AA	NB = 4	AS	NB = 3	PA	NB = 0
		B = 0		B = 1		B = 4
S2	AS	NB = 0	PA	NB = 1	AA	NB = 3
		B = 4		B = 3		B = 0
S3	PA	NB = 0	AA	NB = 3	AS	NB = 2
		B = 4		B = 0		B = 1

^a^Sequence by which steers (4 within each sequence) entered 3 periods of the experiment.

^b^Within each period, steers were subjected to one of the three treatment groups including (1) pure alfalfa pasture (PA), (2) pure alfalfa pasture supplemented with ALFASURE (AA), and (3) alfalfa – sainfoin mixed pasture (AS). Prior to the experiment and during the intervals between each period, steers were fed dry alfalfa hay for 7 days (baseline diet).

^c^Bloat status indicates the number of steers within each treatment groups that either developed clinical bloat (B; bloat scores 1–3) or were non-bloated (NB; bloat score 0).

Illumina paired-end sequencing of the V3-V4 hypervariable region of the 16S rRNA gene generated an average of 48,262(SD = 14,099), 45,541(SD = 16,139), and 42,544(SD = 17,414) high quality sequences per rumen liquid, rumen solid, and fecal sample, respectively. At 97% similarity threshold, alignment of non-singletone OTUs to the Greengenes database resulted in classification of OTUs into 17 and 15 different bacterial phyla for rumen and fecal samples, respectively (Supplementary Table 1). Rumen bacterial communities were dominated by Bacteroidetes (61.1 ± 6.5% in rumen liquid and 53 ± 9.9% in rumen solid fraction) and Firmicutes (26.7 ± 6.9% in rumen liquid and 33.9 ± 8.3% in rumen solid fraction), followed by Fibrobacteres (4.4 ± 2.9% in rumen liquid and 5.9 ± 3.7% in rumen solid fraction), Spirochaetes (1.5 ± 0.8% in rumen liquid and 2.2 ± 1.2% in rumen solid fraction) and Proteobacteria (0.84 ± 0.42% in rumen liquid and 0.54 ± 0.32% in rumen solid fraction). Fecal samples were dominated by Firmicutes (63.36 ± 4.9%) and Bacteroidetes (28.51 ± 4.4%), followed by Spirochaetes (0.90 ± 0.87%) Proteobacteria (0.83 ± 0.30%) and Tenericutes (0.34 ± 0.18%).

### Impact of dietary interventions on biodiversity of rumen microbiota

**Figure 1:**
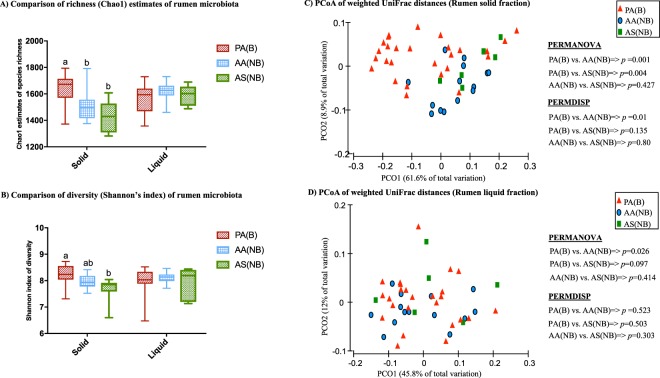
Comparison of diversity metrics of microbial communities. (**A**,**B**) Comparison of within community richness (Chao1 estimates), and diversity (Shannon’s index) of the microbiota of liquid and solid fraction of rumen contents among treatment groups. Box-Whisker plots show average values of richness and diversity: boxes denote interquartile range with a line at the median, whiskers indicate minimal and maximal values, and error bars indicate the standard error for each treatment group. Different superscripts denotes statistical significance among group means (P < 0.05). (**C**,**D**) Principal coordinate analysis (PCoA): comparison of between community diversity based on weighted UniFrac distances of microbial communities in solid and liquid fractions of rumen content. P-value for each comparison was obtained from PERMANOVA and considered significant at P < 0.05. Color codes have been assigned to differentiate between treatment groups: red indicates samples obtained from steers grazed on pure alfalfa and developed clinical bloat (PA(B)), blue indicates samples obtained from non-bloated steers grazed on pure alfalfa supplemented with ALFASURE (AA(NB)), and green indicates samples obtained from non-bloated steers grazed on mixed pastures of pure alfalfa and sainfoin (AS(NB)). Bloat status was indicated with B (developed clinical bloat; bloat scores 1–3) or NB (non-bloated; bloat score 0).

Comparisons of alpha-diversity indices were performed at an even depth of 22,000 sequences per sample. Figure 1A,B compare richness and diversity of rumen microbiota among bloated steers grazed on pure alfalfa pasture and non-bloated steers that received ALFASURE or grazed alfalfa – sainfoin pastures. In general, the solid fraction of rumen content was found to be more affected by treatment. Rumen microbiota of bloated steers showed greater richness as compared to those that did not bloat in ALFASURE and alfalfa – sainfoin groups, while the Shannon’s diversity index of bloated cattle was higher (*P* < 005) than that of steers that did not bloat while grazing alfalfa – sainfoin pasture (Fig. 1A,B). There was no significant difference between the richness and diversity of liquid fractions among treatments. Similarly, comparison of UniFrac distances among treatment groups also revealed that the microbiota of solid rumen content of bloated steers grazing pure alfalfa clustered distinctly from steers that did not bloat while receiving ALFASURE (*p*_(PERMANOVA)_ = 0.001) or grazing alfalfa – sainfoin (*p*_(PERMANOVA)_ = 0.004). Microbiota of solid rumen content of bloated steers were also phylogentically more dispersed (*p*_(PERMDISP)_ = 0.01) compared to non-bloated steers receiving ALFASURE. Within the liquid fraction of the rumen content, microbiota composition only differed between steers that bloated while grazing alfalfa and those that did not bloat with ALFASURE (*p*_(PERMANOVA)_ = 0.026). Within both solid and liquid fractions of the rumen, no clustering pattern was observed between the microbiota of steers that did not bloat as a result of receiving ALFASURE or grazing alfalfa – sainfoin (Fig. 1C,D). To test whether the duration of washout phase (i.e. 7 days) in our crossover experimental design had been long enough to prevent carry-over effect of treatments between subsequent periods, a PERMANOVA model was defined independent of the incidence of bloat to check for the effect of dietary treatments, periods, and their interaction on the composition of rumen microbiota. While microbiota composition of rumen content were significantly affected by treatment (*p*_(rumen-liquid)_ = 0.003 and *p*_(rumen-solid)_ < 0.001) and period (*p*_(rumen-liquid)_ = 0.004 and *p*_(rumen-solid)_ < 0.001), the interaction of treatment and period was not significant for microbiota in either fraction of the rumen content (*p*_(rumen-liquid)_ = 0.447 and *p*_(rumen-solid)_ = 0.072; Supplementary Table S2).

**Figure 2:**
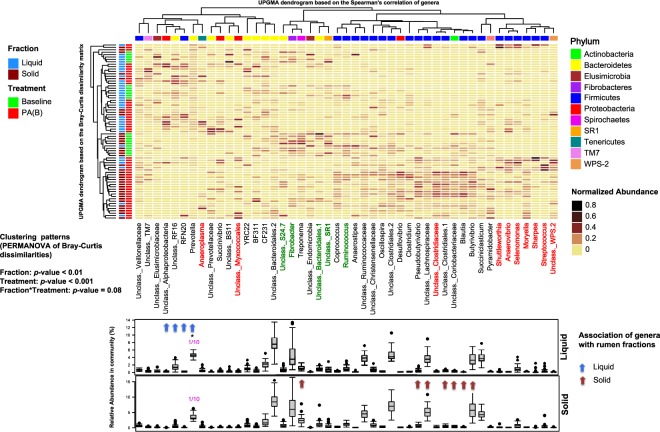
Unsupervised cluster analysis of rumen microbial communities. Rows correspond to samples and columns correspond to abundant genera (>0.1% of community). The “Normalized Abundance” key relates colors to the normalized proportions of genera (relative abundance of each genus divided by the Euclidean length of the column vector). The left dendogram shows clustering pattern of samples based on Bray–Curtis dissimilarities (using unweighted pair group method with arithmetic averaging (UPGMA)). The significance of clustering patterns was calculated based on 9999 permutations and p-values calculated based on PERMANOVA. The top dendogram shows correlation (cooccurrence) of genera based on Spearman’s correlation coefficient. The “Phylum” key relates the top annotations to the corresponding phylum of each genus. The “Fraction” and “Treatment” keys relate samples to their originating rumen fraction (liquid or solid) and treatments groups (baseline: steers grazed on alfalfa hay prior to introduction to alfalfa pasture, PA(B): steers grazed on pure alfalfa pasture and developed clinical bloat). The bottom box-plots show relative abundances of genera in liquid (top) and solid (bottom) fractions. Color codes have been used to highlight association of bacterial genera with different treatment groups (identified using MaAsLin; red indicates significant association with PA(B) and green indicates significant association with baseline). *Indicates that the relative abundance of genus Prevotella has been scaled to 1/10 in order to assist visualization of other abundant genera.

Unsupervised cluster analysis based on the proportion of main bacterial genera (>0.1% of community) revealed that the microbiota of rumen samples from steers that bloated as result of grazing pure alfalfa clustered distinctly (*p*_(PERMANOVA)_ = 0.001) from the microbiota of rumen samples collected during the baseline adaptation phase (steers fed dry alfalfa hay). The same analysis also revealed distinct clustering patterns (*p*_(PERMANOVA)_ = 0.01) between the microbiota of solid and liquid fractions of rumen contents (Fig. 2).

### Association of rumen microbiome with dietary treatment and bloat incidence

**Figure 3:**
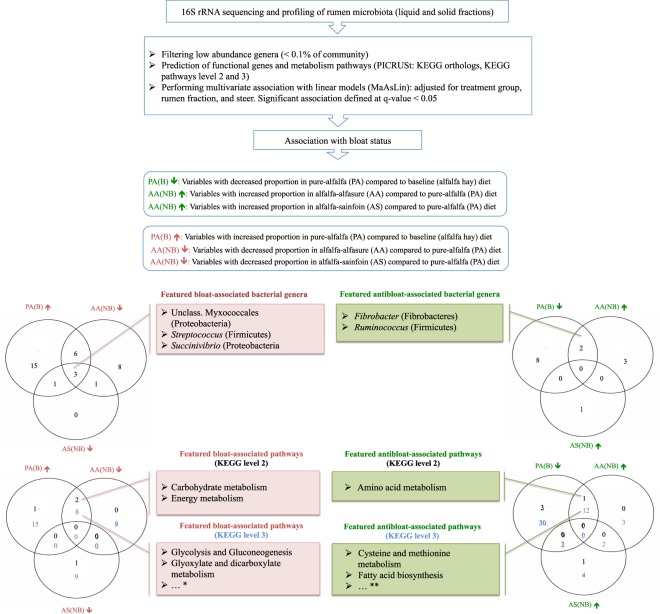
Schematic diagram of data analysis and statistical approaches for identifying feature bacterial genera and functional pathways (KEGG level 2 and 3) associated with bloat status. Abbreviations are used to define treatment groups; PA(B): steers grazed on pure alfalfa pasture and developed clinical bloat; AA(NB): non-bloated steers grazed on pure alfalfa pasture supplemented with ALFASURE; AS(NB) non-bloated steers grazed on mixed pastures of pure alfalfa and sainfoin (AS(NB)). Bloat status was indicated with B (developed clinical bloat; bloat scores 1–3) or NB (non-bloated; bloat score 0). *See Supplementary Table S4 for summary statistics of associative analyses and a complete list of shared/exclusive bacterial genera and KEGG pathways among treatment groups.

Comparison of the main bacterial genera between the microbiota of rumen samples from steers that bloated on pure alfalfa and those collected during the baseline adaptation phase revealed that several co-occurring genera within phyla Firmicutes (including *Streptococcus*, *Selenomonas*, *Sharpea*, *Shuttleworthia* and unclassified Clostridiaceae), Proteobacteria (including unclassified Myxococcales), and Tenericutes (*Anaeroplasma*) were overrepresented, whereas other genera within phyla Bacteroidetes (including unclassified members of the order Bacteroidales and family S24.7), Fibrobacteres (genus *Fibrobacter*), and Firmicutes (genus *Ruminococcus*) were overrepresented in the rumen of steers during the baseline adaptation phase (Fig. 2 and Supplementary Table S3). To further determine key bacterial genera and functional genes that were associated with the occurrence of bloat or lack thereof, the proportion of abundant rumen genera and functional pathways (KEGG level 2 and 3) were compared among treatment groups (Fig. 3). The feature bacterial genera that were found to be consistently overrepresented within the rumen microbiota of bloated steers included unclassified Myxococcales and *Succinivibrio* (within phylum Proteobacteria), and *Streptococcus* (phylum Firmicutes), whereas *Fibrobacter* and *Ruminococcus* were overrepresented in the rumen of non-bloated steers. With regards to predicted functional pathways, in general, carbohydrate metabolism pathways (e.g., glycolysis and gluconeogenesis) were significantly enriched within the rumen microbiota of bloated steers, whereas amino acid metabolism pathways, including cysteine and methionine metabolism were enriched within the rumen microbiota of non-bloated steers (Fig. 3 and Supplementary Table S3).

MaAsLin was also used to explore the association of abundant bacterial genera within solid and liquid rumen fractions independent of treatments. Most of the genera that were significantly associated with solid fraction of rumen contents belonged to the phylum Firmicutes, including *Butyrivibrio*, *Blautia*, *Pseudobutyrivibrio*, and unclassified members of the families Lachnospiraceae and Clostridiaceae. In contrast, genera within the phyla Bacteroidetes (including *Prevotella* and unclassified members of family RF16), and Proteobacteria (unclassified Alphaproteobacteria) were significantly overrepresented within the microbiota of the liquid fraction of rumen contents (Fig. 2).

**Figure 4:**
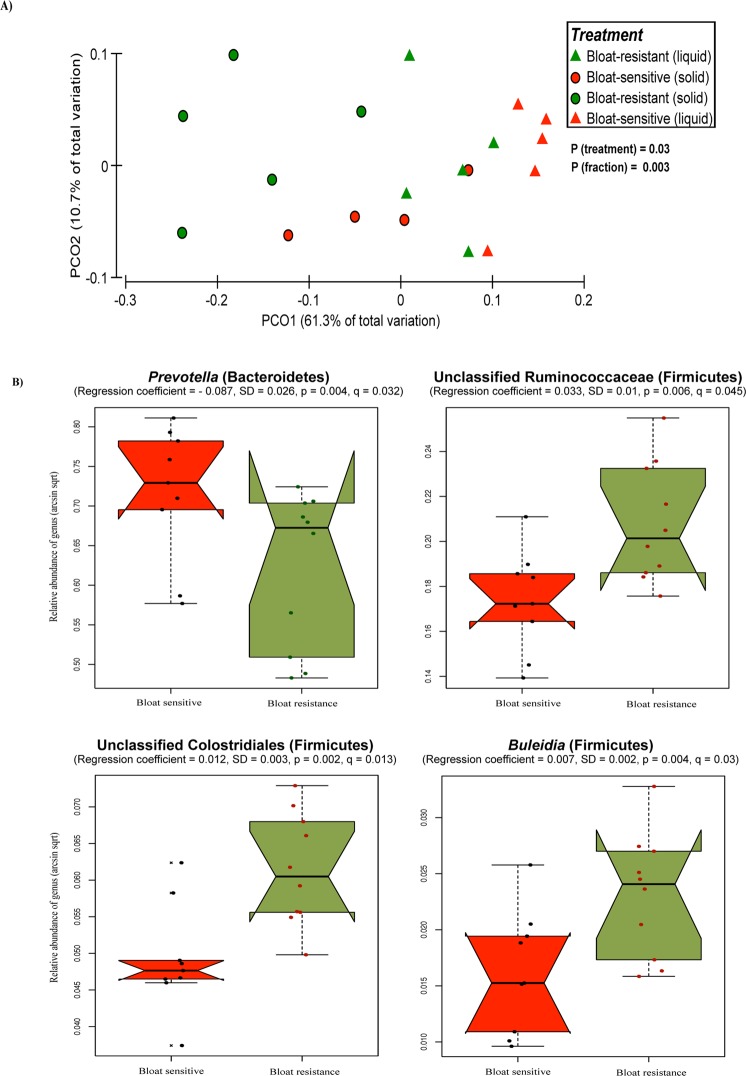
Characterization of bloat-sensitive and bloat-resistant rumen microbiota. (**A**) Principal coordinates analysis (PCoA) of weighted UniFrac distances: between community diversity was compared to test for different clustering patterns of baseline rumen microbiota (samples obtained during baseline adaptation when steers were fed dry alfalfa hay prior to grazing alfalfa pasture) from bloat-sensitive (steers that developed bloat on mixed pasture of alfalfa and sainfoin) and bloat resistant (steers that did not develop bloat on mixed pasture of alfalfa and sainfoin). Color codes have been assigned to differentiate between bloat-sensitive (red) and non-sensitive (green) steers. Triangles denotes samples from the liquid fraction of rumen digesta and circles denote samples obtained from solid fraction. p-value for each comparison was obtained from PERMANOVA and considered significant at P < 0.05. (**B**) Association of bacterial genera with treatment groups: box plot of relative abundances of genera (arcsin square root transformed) indicate the median (horizontal solid line), interquartile range between the first and third quartiles (box), variability outside the upper and lower quartiles (whiskers), and outliers. Color codes indicate the treatment groups: red indicates samples obtained from bloat-sensitive animals and green indicates samples obtained from bloat-resistant animals.

Finally, the baseline rumen microbiome of steers that did not bloat while grazing alfalfa – sainfoin pasture were compared to those that did bloat by comparison of beta-diversity based on UniFrac distances, revealing distinct clustering patterns between the two groups of steers (*p*_(PERMANOVA)_ = 0.03; Fig. 4A). Comparison of abundant bacterial genera between the two groups also revealed overrepresentation of several Firmicutes genera, including *Buleidia*, unclassified Ruminococcaceae, and unclassified Colostridiales, within the rumen microbiota of steers that did not bloat, whereas *Prevotella* was significantly overrepresented in the baseline rumen microbiota of bloated steers (Fig. 4B).

### The impact of dietary treatments and bloat incidence on fecal microbiota

**Figure 5:**
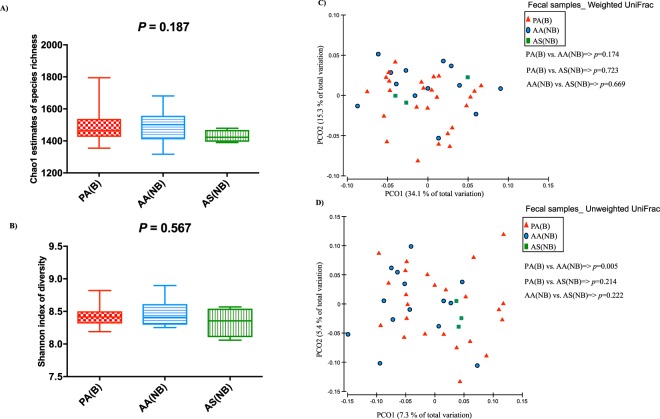
Comparison of biodiversity metrics of fecal microbial communities. (**A**,**B**) Comparison of within community richness (Chao1 estimates) and diversity (Shannon’s index) among treatment groups. Box-Whisker plots show average values of richness and diversity: boxes denote interquartile range with a line at the median, whiskers indicate minimal and maximal values, and error bars indicate the standard error for each treatment group. P-value for each comparison was obtained from ANOVA (SAS 9.3) and considered significant at P < 0.05. (**C**,**D**) Principal coordinate analysis (PCoA): comparison of between community diversity based on weighted and unweighted UniFrac distances of fecal microbial communities. P-value for each comparison was obtained from PERMANOVA and considered significant at P < 0.05. Color codes have been assigned to differentiate between treatment groups: red indicates samples obtained from steers grazed on pure alfalfa and developed clinical bloat (PA(B)), blue indicates samples obtained from nonbloated steers grazed on pure alfalfa supplemented with ALFASURE (AA(NB)), and green indicates samples obtained from non-bloated steers grazed on mixed pastures of pure alfalfa and sainfoin (AS(NB)). Bloat status was indicated with B (developed clinical bloat; bloat scores 1–3) or NB (non-bloated; bloat score 0).

**Figure 6:**
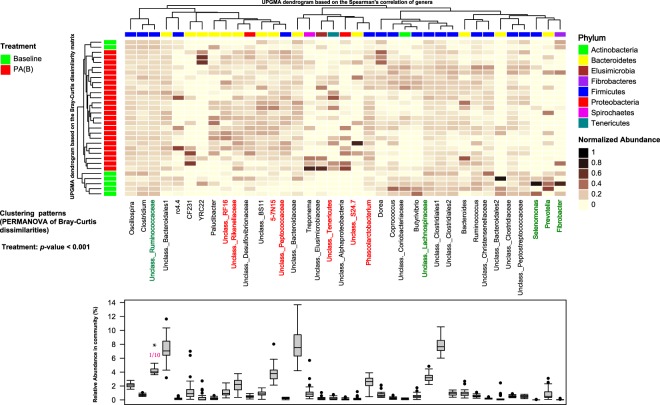
Unsupervised cluster analysis of fecal microbial communities. Rows correspond to samples and columns correspond to abundant genera (>0.1% of community). The “Normalized Abundance” key relates colors to the normalized proportions of genera (relative abundance of each genus divided by the Euclidean length of the column vector). The left dendogram shows clustering pattern of samples based on Bray–Curtis dissimilarities (using unweighted pair group method with arithmetic averaging (UPGMA)). The significance of clustering patterns was calculated based on 9999 permutations and p-values calculated based on PERMANOVA. The top dendogram shows correlation (co-occurrence) of genera based on Spearman’s correlation coefficient. The “Phylum” key relates the top annotations to the corresponding phylum of each genus. The “Treatment” key relates samples to treatments groups (baseline: steers grazed on alfalfa hay prior to introduction to alfalfa pasture, PA(B): steers grazed on pure alfalfa pasture and developed clinical bloat). The bottom box-plots show the relative abundances of genera. The associations of genera with treatment groups were identified using MaAsLin, considering a significance cut-off q-value of 0.05 and adjusted for potential confounders including treatment groups and subject (steers)). Color codes have been used to highlight bacterial genera that were associated with treatment groups (red: significant association with PA(B); q-value < 0.05 and green: significant association with baseline; q-value < 0.05). *The relative abundance of Unclassified Ruminococcaceae has been scaled to 1/10 in order to assist visualization of other genera.

Unlike rumen microbiota, the richness and diversity of fecal microbiota was not affected by treatments (Fig. 5A,B). With respect to the beta-diversity of fecal microbiota, only unweighted UniFrac distances differed (*p*_(PERMANOVA)_ = 0.005) between the microbiota of steers that bloated grazing alfalfa and those that received ALFASURE. In addition, unsupervised clustering analysis based on the proportions of main bacterial genera (>0.1% of community) revealed a distinct clustering pattern between the microbiota of fecal samples collected during the baseline adaptation phase and those that bloated while grazing alfalfa (*p*_(PERMANOVA)_ < 0.001; Fig. 6). *Phascolarctobacterium* and unclassified Peptococcaceae within the phylum Firmicutes, unclassified Bacteroidetes belonging to families Rikenellaceae, S24-7, and RF16 were overrepresented within the fecal microbiota of bloated steers grazing alfalfa, whereas *Fibrobacter*, *Prevotella*, *Selenomonas* and unclassified Lachnospiraceae were overrepresented within the fecal microbiota of samples collected during the baseline adaptation phase.

## Discussion

Composition and functionality of rumen microbiota can be influenced by a wide array of host and environmental factors among which diet appears to play a central role in shaping the overall profile of rumen microbial ecosystem^17^. In beef cattle, grazing of wheat and alfalfa pastures has been associated with frothy bloat^1,9^, with recent evidence suggesting that this condition may be linked to a perturbed rumen microbiome in cattle grazing wheat pastures^18^. In the present study, we showed that transition from alfalfa hay to alfalfa pasture resulted in rapid development of frothy bloat in the rumen ecosystem of steers. We further demonstrated that bloat preventive strategies, in particular supplementation of drinking water with ALFASURE reduces the development of alfalfa-induced frothy bloat. ALFASURE is a pluronic detergent known to prevent pasture bloat via reducing rumen fluid viscosity and disrupting the stability of the froth that causes bloat. This may be in part due to the ability of pluronic detergents to prevent the formation of micelle within the rumen ecosystem, which in turn can disrupt gas bubbles and prevent them from coalescing^19^. Sainfoin is a bloat-safe forage that contains condensed tannins^20^. Tannins are a family of polyphenolic compounds capable of binding to soluble proteins, polysaccharides and other macromolecules^21^. Thus, by binding to plant proteins and preventing them from being solubilized into the ruminal fluid, condensed tannins of sainfoin can prevent the formation of proteinaceous gas-trapping foam within rumen contents and therefore prevent frothy bloat. It has been demonstrated that inclusion of sainfoin with alfalfa at a concentration of 10–12% (DM basis) reduces the incidence of bloat^22,23^.

### Bloat-associated shifts in diversity and composition of rumen microbiota

Transition from alfalfa hay to alfalfa pasture resulted in increased richness and diversity of the rumen microbiota. Increased microbial diversity is an intrinsic characteristic of biofilm formation and proliferation; a principle referred to as “insurance hypothesis” and known to be the predominant growth mode for microbial communities in natural environments^24,25^. Availability of a diverse genetic pool allows associated microbial consortia to form structured communities - embedded in extracellular matrices - where end products produced by one colony can be sequentially used by closely associated colonies^25,26^. In the current study, increased diversity of the rumen microbiota of bloated steers was concomitant with enrichment of predicted bacterial genes that are responsible for metabolism of complex carbohydrates. This might be indicative of the robustness of ruminal biofilm for degrading complex plant polysaccharides and production of the exopolysaccharide slime that is associated with bloat^1^. Further, we observed that increased microbiota diversity in bloated animals was more pronounced in the solid fraction of rumen contents. One possible explanation could be that in the bloated rumen ecosystem, increased viscosity results in entrapment of end products (metabolites) of the enzymatic degradation of plant polysaccharides surrounding solid particles, which in turn may result in uncontrolled proliferation of solid-associated biofilms.

Transition from hay to alfalfa-pasture also resulted in sharp compositional shifts in the rumen microbiota of grazing steers. In particular, our associative analysis revealed that a group of co-occurring bacterial genera within the phylum Firmicutes - including *Streptococcus*, *Selenomonas*, *Shuttleworthia* and unclassified Clostridiaceae - were enriched within the rumen contents of bloated steers; whereas other genera such as *Ruminococcus* and *Fibrobacter* were associated with baseline alfalfa hay diet. *Ruminococcus* spp. (e.g. *R*. *flavefaciens* and *R*. *albus*) and *Fibrobacter succinogenes* are commonly regarded as the main cellulose-degrading species within the rumen ecosystem^27^ and are predominantly present in high fiber diets^28^. In contrast, *Streptococcus* and *Selenomonas* are dominant bacterial genera within rumen microbiota of ruminants receiving high soluble carbohydrate diets^28,29^ or high quality fresh forage diets with high levels of soluble protein^30^. Some species of these genera, including *Streptococcus bovis* and *Selenomonas ruminantium*, are known to produce and store high amounts of reserve polysaccharides in their cytoplasm which can serve as precursors for extracellular polymeric substances of biofilms^31,32^. Previously, Min *et al*.^33^ reported the *in vitro* ability of *S*. *bovis* to actively produce biofilms. This same study also revealed increased *in vivo* biofilm production in rumen ecosystem of steers exposed to wheat forage. Unfortunately, the DGGE approached used by Min *et al*.^33^ lacked the required resolution for accurate classification of bacterial genera and their association with bloat.

By exploring the dynamics of rumen microbiome during development of wheat-induced frothy bloat, Pitta *et al*.^18^ observed that *Ruminococcus*, *Lactobacillus*, and *Prevotella* were overrepresented in normal rumen contents, whereas *Clostridium*, *Eubacterium*, and *Butyrivibrio* were enriched within the rumen contents of bloated cattle. The authors concluded that the latter group of bacteria can utilize the oligosaccharides trapped in the biofilm, which they hypothesized could be related to the high proportion of genes within these Firmicutes that contribute to carbohydrate metabolism. In the present study, we observed that bloat-associated enrichment of *Streptococcus*, *Selenomonas*, *Shuttleworthia* and unclassified Clostridiaceae was associated with increased proportion of predicted microbial genes that were annotated to carbohydrate metabolism pathways. These findings led us to speculate that bloat-associated biofilms naturally select for genera that have higher potential for rapid metabolism of soluble carbohydrates trapped within exopolysaccharide slime. Ironically, Pitta *et al*.^18^ reported that the abundance and diversity of Carbohydrate Active Enzymes (CAZy) was greatly reduced in the rumen content of bloated steers on wheat pasture. Discrepancies between our observations and those of Pitta *et al*.^18^ might have been in part due to possible differences in the etiology and pathogenesis of alfalfa-induced versus wheat-induced frothy bloat, or perhaps because of methodological differences between the two studies. However, it should be noted that metagenomics approaches, either based on whole-genome sequencing or predicted from marker genes, face fundamental limitations to directly reflect the functional activities of microbial communities. Indeed, parallel metagenomics and metatransciptomics investigation of human gut microbiome has revealed that certain microbial clades possess metabolic activities that tend to be consistently overexpressed (mRNA is found to be more abundant than the equivalent DNA); whereas other genes can be consistently under expressed within microbial communities (DNA is found to be more abundant than the equivalent mRNA). This demonstrates that the translational activity of a given metagenome is highly dynamic and largely regulated by metabolome profile (i.e. availability/deficiency of certain metabolites in the ecosystem)^34^.

### Identification of feature bacterial genera associated with development of frothy bloat

Multiple comparisons among the composition of rumen microbiota of bloated steers with those that did not develop bloat following dietary interventions led us to identify key bacterial genera that were consistently associated with development of frothy bloat. In particular, we observed that *Streptococcus*, *Succinivibrio*, and Unclass. Myxococcales were consistently enriched in the rumen ecosystem of bloated steers, whereas, *Fibrobacter* and *Ruminococcus* were overrepresented in the rumen contents of non-bloated steers. Previously, Pitta *et al*.^18^ also observed that the proportion of *Ruminococcus* was decreased in the rumen content of bloated steers grazing wheat pasture. This, together with our observation regarding simultaneous decreases in proportions of *Fibrobacter* and *Ruminococcus*, suggests that development of frothy bloat disfavors the growth of the main fiber-degrading bacterial lineages. On the other hand, we observed that members of the phylum Proteobacteria, including *Succinivibrio* and Myxococcales were enriched in the rumen content of bloated steers. Proteobacteria have been reported to be enriched during adaptation to high-grain diets^35,36^ and appear to be more tolerant of low rumen pH^37^. Pitta *et al*.^18^ also observed increased proportion of the phylum Proteobacteria in the rumen content of bloated steers on wheat pastures. Simultaneous DNA and RNA analyses of rumen microbiome of steers fed grain-based diet^38^ suggested that metabolic contributions of Proteobacteria are greater than their proportion in rumen community. The metabolic activity of family Myxococcales in the rumen ecosystem is unknown. However, *Succinivibrio* spp. have been identified as part of the core-rumen microbiota of cattle which predominate during adaptation to high starch diets^37,39^. *Succinivibrio dextrinosolvens*, is a predominant rumen dweller that contributes to fermentation of a variety of carbohydrates and its end products (succinate and formate) can serve as intermediates of rumen fermentation that are further used by other microorganisms^39,40^. Our results suggest that Proteobacteria play a central role in development of alfalfa-induced frothy bloat, likely via contribution to rapid metabolism of complex polysaccharides.

An important caveat of the present study was the relatively short washout phase (i.e. 7 days) between different treatment periods of our crossover experimental design, which might have resulted in carryover effect of previous treatments and limit the ability of different groups of microbes to proliferate and establish stable populations following introduction to new dietary regimens. Despite this shortcoming, our results suggested that some steers were more susceptible to development of frothy bloat when grazing mixed alfalfa – sainfoin pastures. Comparison of the baseline rumen microbiota of this group of steers with those that were resistant to development of bloat when grazing on mixed alfalfa – sainfoin pastures revealed distinct clustering patterns. In particular, we observed that the proportion of *Prevotella* was significantly higher in the baseline rumen microbiota of bloat-sensitive steers. *Prevotella* spp. are predominant rumen dwellers^41,42^ which possess extensive amylolytic and proteolytic activities^43,44^. Overrepresentation of *Prevotella* in the rumen microbiota of bloat-sensitive steers led us to speculate that the metabolic activity and end/by products of this bacterial lineage may accelerate the formation and proliferation of bloat-associated biofilms.

### Bloat-associated shifts in the composition of hindgut microbiota

The bypass of undigested rumen contents to the intestines can modify fermentation profile and microbiota composition of the hindgut ecosystem^45–47^. In the present study, transition from baseline diet to alfalfa pasture was associated with moderate shifts in the composition of fecal microbiota of steers. More specifically, we observed an underrepresentation of fiber degrading bacterial lineages such as *Fibrobacter* and unclassified Ruminococcaceae in the fecal microbiota of bloated steers, which might be indicative of disrupted fermentation profile of the hindgut ecosystem as a result of frothy bloat. However, we failed to observe any notable influence of dietary interventions on the fecal microbiota of bloated versus non-bloated steers grazed on alfalfa pastures. Comparison of baseline fecal microbiota between bloat-sensitive steers with those that did not develop bloat on mixed alfalfa – sainfoin pastures also did not reveal any significant differences. Overall, our data suggest that rapid screening of fecal microbiota does not offer a viable strategy to identify steers that may be susceptible to development of frothy bloat on alfalfa pastures.

## Conclusions

In the present study, we described bloat-associated shifts in the composition of rumen and fecal bacterial communities of steers grazing alfalfa pastures. Whereas only slight differences existed between the fecal microbiota of bloated versus non-bloated steers, we observed that the development of frothy bloat was associated with dramatic shifts in the diversity of rumen microbiota, particularly those associated with the solid fraction of rumen contents. Increased species-richness and diversity of fiber-associated microbiota suggested that development of frothy bloat might be in part due to the rapid proliferation of fiber-adherent biofilms. In particular, bacterial genera and predicted functional pathways that are associated with metabolism of complex plant polysaccharides were enriched within the rumen content of bloated steers.

## Methods

### Ethics statement

Protocol (1214) for this experiment was reviewed and approved by the Animal Care Committee (ACC) of the Lethbridge Research Centre (LRC), Lethbridge, Alberta. Cattle were cared for according to the guidelines of the Canadian Council on Animal Care (CCAC, 1993).

### Experimental Design and assessment of bloat scores

Twelve mature (3–4 y old) ruminally-fistulated Angus steers were allocated to a 3 × 3 crossover experimental design, subjecting all animals to three different dietary treatments evenly distributed across three time periods. The treatments included: (1) pure alfalfa pasture; steers grazed on pure stands of alfalfa for 6 h where bloat occurred; (2) steers grazed on pure stands of alfalfa for 6 h but treated with the pluronic detergent ALFASURE (0.25 mL L^−1^; Rafter 8 Products Inc., Calgary, AB, Canada) to prevent development of bloat; and (3) steers grazed for 6 h on mixed sainfoin – alfalfa pastures in which a minimum of 15% of the standing pasture dry matter was composed of sainfoin. Within this group, we had two subgroups of (a) steers that did not experience bloat, and (b) steers that did experience clinical bloat. A primary adaptation period (baseline phase) of 3 weeks preceded the experiment during which all steers were fed dry alfalfa hay.

The crossover experiment was conducted in 3 periods each divided into an adaptation/washout phase (7 days: steers fed alfalfa hay) and sampling phase (4 days: steers grazed on pastures). At the start of the experiment, all groups were released at 0830 into either pure vegetative alfalfa (with and without ALFASURE) or mixed vegetative alfalfa – sainfoin pastures. The steers were allowed to freely graze for 6 h and at 1430 all steers were removed from the pasture and retained in a pen overnight without feed, but with free access to water. On pasture, all steers had access to water, but those steers on the ALFASURE treatment had access only to water that contained 0.25 mL/L of ALFASURE, which on average delivered 9.5 g/d of poloxalene to each animal. Steers were closely monitored every 30 min during grazing and for 2 h after grazing to record the incidences of clinical bloat according to the procedure described by Majak *et al*.^48^. In brief, bloat scores were assigned as follows: 0 = Normal (no visible sign of bloat), 1 = Slight (slight distention of the left side of the animal), 2 = Marked: (marked distention of the left side of the animal with asymmetrical (egg-shape) look when walking away from the observer), and 3 = Severe (severe distention above the top of back and inside from right side of the animal). A single steer bloating on one day was counted as one case of bloat. If the assessment indicated that the sum of bloat incidences reached 9 or more in any of the three groups within the sampling phase, then steers were crossed over between pastures and the experiment was repeated until an additional 9 cases of bloat were obtained. For the purpose of this study, rumen and fecal samples collected from steers with a bloat score of 0 were considered as non-bloated and those collected from steers with a bloat score between 1–3 were considered as bloated.

### Rumen sample collection and processing

Prior to the grazing experiment, rumen digesta (from both cranial and caudal ventral sacs of the rumen) and fecal samples were collected from each steer at the end of the primary adaptation period (baseline samples). In period 1, after the steers were introduced to the pasture, rumen digesta and fecal samples were collected from steers on 4 consecutive days, immediately after they were removed from their assigned paddock in the afternoon. The experimental procedure for period 2 and 3 were exactly the same as period 1, but steers were crossed among treatment paddocks.

Approximately 50 g of ruminal digesta was collected from each of the cranial and caudal ventral sacs of the rumen. Digesta was transferred into a heavy-walled 250-mL beaker and squeezed with a Bodum coffee maker plunger (Bodum Inc.,Triengen, Switzerland). Aliquots of fluid digesta (5 mL) were placed in aluminum foil dishes and flash-frozen in liquid nitrogen and stored at −80 °C. Solid residue was suspended in 30 mL cold (4 °C) grinding buffer (100 mM Tris-HCl, 500 mM EDTA, 1.5MNaCl, 1 mg mL#1 proteinase K, pH 8.0). The suspension was placed in a shallow aluminum foil dish and flash frozen in liquid nitrogen. All samples were stored at -80 °C until further processing.

### DNA extraction and quality check

To extract genomic DNA, each frozen sample was coarsely ground under liquid nitrogen in a precooled porcelain mortar. Samples were then transferred into a precooled Retsch RM 100 Mortar Grinder equipped with a stainless-steel mortar bowl and pestle (F. Kurt Retsch GmbH and Co. KG, Haan, Germany) and ground for a further 5 min under liquid nitrogen. Liquid nitrogen was added to the mortar bowl during grinding as needed to maintain the grinding mixture in a semi-fluid state. The ground samples were transferred to a 200-mL wide-mouth centrifuge bottle. The sample was then slowly poured into a 50 mL falcon tube. Genomic DNA was then extracted from 150–250 mg of each sample using QIAamp DNA Stool Mini Kit (Qiagen Inc., Mississauga, ON, Canada). The extracted DNA was quality checked using agarose gel electrophoresis and quantified by Picogreen dsDNA (Invitrogen, Eurgene, OR, USA). DNA samples were then normalized to 20 ng/µL, and quality checked by PCR amplification of the 16S rRNA gene using universal primers 27F (5′-GAAGAGTTTGATCATGGCTCAG-3′) and 342R (5′-CTGCTGCCTCCCGTAG-3′) as described by Khafipour *et al*.^36^. Amplicons were verified by agarose gel electrophoresis.

### Library construction and Illumina sequencing

The V3-V4 region of 16S rRNA gene was targeted for PCR amplification using modified primers as described by Derakhshani *et al*^49^. PCR reaction for each sample was performed in duplicate and contained 1.0 µL of pre-normalized DNA, 1.0 µL of each forward and reverse primers (10 µM), 12 µL HPLC grade water (Fisher Scientific, Ottawa, ON, Canada) and 10 µL 5 Prime Hot MasterMix (5 Prime Inc., Gaithersburg, MD, USA). Reactions consisted of an initial denaturing step at 94 °C for 3 min followed by 35 amplification cycles at 94 °C for 45 sec, 50 °C for 60 sec, and 72 °C for 90 sec; finalized by an extension step at 72 °C for 10 min in an Eppendorf Mastercycler pro (Eppendorf, Hamburg, Germany). PCR products were then purified using ZR-96 DNA Clean-up Kit (ZYMO Research, Irvine, CA, USA) to remove primers, dNTPs and reaction components. The V3-V4 library was then generated by pooling 200 ng of each sample as quantified by Picogreen dsDNA (Invitrogen, Burlington, ON, Canada). This was followed by multiple dilution steps using pre-chilled hybridization buffer (HT1) (Illumina, San Diego, CA, USA) to bring the pooled amplicons to a final concentration of 5 pM, as measured by a Qubit 2.0 Fluorometer (Life technologies, Burlington, ON, Canada). Finally, 15% of PhiX control library was spiked into the amplicon pool to improve the unbalanced and biased base composition, a known characteristic of low diversity 16S rRNA libraries. Customized sequencing primers were synthesized and purified by polyacrylamide gel electrophoresis (Integrated DNA Technologies, Coralville, IA, USA) and added to the MiSeq Reagent Kit V3 (600-cycle) (Illumina, San Diego, CA, USA). The 300 paired-end sequencing reaction was performed on a MiSeq platform (Illumina, San Diego, CA, USA) at the Gut Microbiome and Large Animal Biosecurity Laboratories, Department of Animal Science, University of Manitoba, Canada. The sequencing data are uploaded into the Sequence Read Archive (SRA) of NCBI (http://www.ncbi.nlm.nih.gov/sra) and can be accessed through accession numbers SRR7350213-SRR7350413. Metadata used for bioinformatics and statistical analyses can be found in Supplementary Table S4.

### Bioinformatics and statistical analysis

The PANDAseq assembler^50^ was used to merge and fix the overlapping paired-end Illumina fastq files. All the sequences with low quality base calling scores as well as those containing uncalled bases (N) in the overlapping region were discarded. The output fastq file was then analyzed by downstream computational pipelines of the open source software package QIIME^51^. Assembled reads were demultiplexed according to the barcode sequences, chimeric reads were filtered using UCHIME^52^ and sequences were assigned to Operational Taxonomic Units (OTU) using the QIIME implementation of UCLUST^53^ at a 97% pairwise identity threshold. Taxonomies were assigned to the representative sequence of each OTU using the RDP classifier^54^ and aligned with the Greengenes Core reference database^55^ using PyNAST algorithms^56^. A phylogenetic tree was built with FastTree 2.1.3.^57^ for further comparisons between microbial communities. Within community richness (Chao 1 estimator of species richness) and diversity (Shannon’s index) were calculated using QIIME at an even depth of 22,000 and 8,000 sequences per rumen and fecal samples, respectively. To compare microbial composition among samples, β-diversity was measured by calculating the weighted and unweighted UniFrac distances^58^. Principal coordinate analysis (PCoA) was applied on resulting distance matrices to generate two-dimensional plots using PRIMER v6 software^59^. Finally, open source software PICRUSt (phylogenetic investigation of communities by reconstruction of unobserved states)^60^ was used to predict the functional genes of the classified members of the rumen microbiota, and assign them to corresponding KEGG^61^ orthologs (KOs) and pathways (level 2 and 3).

### Unsupervised clustering analysis

To test for discrete clustering pattern of samples, the relative abundance of the OTUs were binned into genus-level taxonomic groups and filtered to retain the most abundant genera across all samples (cutoff value of >0.1% of community). The resulting relative abundance table was normalized (values divided by the Euclidean length of the row vector) to correct for compositionality and also aide in the heatmap-visualization of differentially abundant genera. The dissimilarity of samples were calculated based on Bray–Curtis measure using the R “vegan” package and the resulting matrix was subjected to unsupervised hierarchical clustering using the R “dendextend” package^62^. The resultant data were visualized over the heatmap of abundance matrix using the R “complexheatmap” package^63^. Genera were also clustered based on their Spearman’s correlation coefficient using R “complexheatmap” package.

### Statistical analysis

The UNIVARIATE procedure of SAS (SAS 9.3, 2012) was used to test the normality of residuals for alpha biodiversity data. Non-normally distributed data were log transformed and then used to assess the effect of MAP infection using the MIXED procedure of SAS. All pairwise comparisons among the groups were tested using Tukey’s studentized range distribution. Permutational multivariate analysis of variance (PERMANOVA)^64^ was used to calculate *P*-values and test for significant differences of β-diversity (Bray-Curtis dissimilarity, weighted and unwieghted UniFrac distance matrices) among treatment groups. Label permutations were used in PERMANOVA to estimate the distribution of test statistics under the null hypothesis that within-group distances are not significantly different from between-group distances^65^.

Multivariate analysis with linear modeling (MaAsLin^66^) was used to determine significant associations of bacterial genera/functional pathways with treatment groups. MaAsLin included a general linear model with treatment groups as categorical predictor variables and arcsine-square root transformed relative abundances of bacterial genera/functional pathways as the response variable. In addition, MaAsLin also accounted for other potential confounders (covariates) that could affect the profile of microbiota, including rumen fraction (liquid and solid), period, and subject (steers). Multiple hypotheses were adjusted by Benjamini and Hochberg false discovery rate (FDR). Unless otherwise indicated, significant associations were considered below a *q*-value threshold of 0.05.

## Supplementary information

Supplementary Table S1

Supplementary Table S2

Supplementary Table S3

Supplementary Table S4
